# M2 polarization enhances silica nanoparticle uptake by macrophages

**DOI:** 10.3389/fphar.2015.00055

**Published:** 2015-03-23

**Authors:** Jessica Hoppstädter, Michelle Seif, Anna Dembek, Christian Cavelius, Hanno Huwer, Annette Kraegeloh, Alexandra K. Kiemer

**Affiliations:** ^1^Department of Pharmacy, Pharmaceutical Biology, Saarland University, SaarbrueckenGermany; ^2^Korea Institute of Science and Technology Europe, SaarbrueckenGermany; ^3^Nano Cell Interactions Group, INM – Leibniz Institute for New Materials, SaarbrueckenGermany; ^4^Department of Cardiothoracic Surgery, Voelklingen Heart Centre, VoelklingenGermany

**Keywords:** inflammation, mononuclear phagocyte system, phagocytosis, endocytosis, lung macrophages, alveolar macrophage, tumor-associated macrophage, real-time RT-PCR

## Abstract

While silica nanoparticles have enabled numerous industrial and medical applications, their toxicological safety requires further evaluation. Macrophages are the major cell population responsible for nanoparticle clearance *in vivo*. The prevailing macrophage phenotype largely depends on the local immune status of the host. Whereas M1-polarized macrophages are considered as pro-inflammatory macrophages involved in host defense, M2 macrophages exhibit anti-inflammatory and wound-healing properties, but also promote tumor growth. We employed different models of M1 and M2 polarization: granulocyte-macrophage colony-stimulating factor/lipopolysaccharide (LPS)/interferon (IFN)-γ was used to generate primary human M1 cells and macrophage colony-stimulating factor (M-CSF)/interleukin (IL)-10 to differentiate M2 monocyte-derived macrophages (MDM). PMA-differentiated THP-1 cells were polarized towards an M1 type by LPS/IFN-γ and towards M2 by IL-10. Uptake of fluorescent silica nanoparticles (Ø26 and 41 nm) and microparticles (Ø1.75 μm) was quantified. At the concentration used (50 μg/ml), silica nanoparticles did not influence cell viability as assessed by MTT assay. Nanoparticle uptake was enhanced in M2-polarized primary human MDM compared with M1 cells, as shown by flow cytometric and microscopic approaches. In contrast, the uptake of microparticles did not differ between M1 and M2 phenotypes. M2 polarization was also associated with increased nanoparticle uptake in the macrophage-like THP-1 cell line. In accordance, *in vivo* polarized M2-like primary human tumor-associated macrophages obtained from lung tumors took up more nanoparticles than M1-like alveolar macrophages isolated from the surrounding lung tissue. In summary, our data indicate that the M2 polarization of macrophages promotes nanoparticle internalization. Therefore, the phenotypical differences between macrophage subsets should be taken into consideration in future investigations on nanosafety, but might also open up therapeutic perspectives allowing to specifically target M2 polarized macrophages.

## Introduction

Numerous types of nanomaterials, such as quantum dots or silica, carbon, zinc oxide, and gold nanoparticles, have been shown to induce inflammatory responses both *in vitro* and *in vivo* (Deng et al., 2011; Autengruber et al., 2014; Kusaka et al., 2014; Roy et al., 2014; Wu and Tang, 2014). Macrophages represent critical regulators of inflammatory processes and also exhibit a high uptake potential for nanoparticles (Sica and Mantovani, 2012; Diesel et al., 2013; Klein et al., 2013; Amoozgar and Goldberg, 2014; Kusaka et al., 2014). Therefore, the investigation of macrophage responses upon nanoparticle exposure is highly relevant for the prediction of potentially harmful effects.

Most cellular models used so far to investigate nanoparticle-associated inflammation do not take macrophage heterogeneity into account. A study by Jones et al. (2013) recently reported that the rate of nanoparticle clearance *in vivo* differs largely between mouse strains dependent on their preference for either Th1- or Th2-responses. C57BL/6 mice preferentially produce T helper type 1 (Th1) cytokines, such as interferon (IFN)-γ, whereas those from Balb/c mice favor T helper type 2 (Th2) cytokine production, e.g., interleukin (IL)-10. In addition to their distinct T-cell responses, *in vitro* investigations have demonstrated that macrophages from these mouse strains exert different reactions in response to the bacterial cell wall component and activator of the innate immune response lipopolysaccharide (LPS; Watanabe et al., 2004).

With reference to Th1/Th2 polarization, two distinct states of polarized activation for macrophages have been suggested: the classically activated (M1) macrophage phenotype and the alternatively activated (M2) macrophage phenotype. M1 macrophages act as effector cells in Th1 responses, whereas M2 macrophages appear to be involved in immunosuppression and tissue repair. LPS and the Th1 cytokine IFN-γ polarize macrophages towards the M1 phenotype associated with the production of large amounts of pro-inflammatory mediators, such as tumor-necrosis factor (TNF)-α, nitric oxide, IL-12, and IL-23, thereby promoting pathogen clearance and antigen specific Th1 and Th17 cell responses. In contrast, exposure of macrophages to the Th2 cytokines IL-4 or IL-10 induces an M2 phenotype characterized by the production of high levels of IL-10 and IL-1 receptor antagonist and low expression of IL-12. These cells facilitate parasite clearance and reduce inflammation, but are also considered to contribute to asthma exacerbations and tumor progression (Gordon and Mantovani, 2011; Sica and Mantovani, 2012; Boorsma et al., 2013; Mills and Ley, 2014).

Using depletion strategies, Jones et al. (2013) demonstrated that macrophages are involved in the enhanced clearance of 300 nm cylindrical PEG hydrogel nanoparticles observed in Th2-prone mice. In accordance, macrophages isolated from Th1 strains showed a lower capacity than macrophages from Th2 strains to take up these nanoparticles. *In vitro* polarization led to similar results, suggesting that macrophage polarization critically affects nanoparticle uptake.

Other factors influencing cellular uptake include nanoparticle morphology, i.e., size and shape, and the materials used (Albanese et al., 2012; Kusaka et al., 2014; Truong et al., 2014). Therefore, the findings by Jones et al. (2013) might not apply to other types of nanoparticles.

Among different nanomaterials, silica nanoparticles are widely used in various applications, ranging from additives for plastics or food to targeted drug carrier systems. Worldwide, 1.5 million tons of amorphous silica nanoparticles are produced annually. This huge production rate is even expected to rise due to growth sectors such as energy and information technology as well as nanomedicine (BMBF, 2013).

Despite the increasing number of applications for silica nanoparticles, the influence of macrophage polarization on their uptake and thereby their clearance has not been characterized yet. Thus, we examined the uptake potential of differentially polarized human macrophages for silica nanoparticles by employing fluorescently labeled particles.

## Materials and Methods

### Cell Culture

#### Human Monocyte-Derived Macrophages (MDM)

Buffy coats were obtained from healthy adult blood donors (Blood Donation Center, Saarbrücken, Germany). The use of human material for the isolation of primary cells was approved by the local ethics committee (State Medical Board of Registration, Saarland, Germany; permission no. 130/08). Monocytes were isolated from buffy coats with CD14 microbeads (Miltenyi Biotec) as suggested by the supplier. In brief, peripheral blood mononuclear cells (PBMC) were isolated by density gradient centrifugation using Pancoll (PAN Biotech). PBMC were washed in PBS (phosphate buffered saline, Sigma–Aldrich) containing EDTA (2 mM, Sigma–Aldrich) and remaining erythrocytes were lysed in BD Pharm Lyse (BD Biosciences). After washing twice with PBS/EDTA, monocytes were purified from PBMC using magnetic cell sorting with anti-CD14 microbeads (Miltenyi Biotec) according to the manufacturer’s instructions, except that FCS was used instead of BSA to prepare respective buffers. Monocyte purity was >95% as assessed by CD14 expression (data not shown).

For macrophage polarization, monocytes were cultured in 12-well plates at a density of 0.5 × 10^6^ cells per well for 5 days at 37°C and 5% CO_2_ in Macrophage-SFM (Life Technologies) supplemented with either 10 ng/ml human recombinant macrophage colony-stimulating factor (M-CSF) or granulocyte-macrophage colony-stimulating factor (GM-CSF; Miltenyi Biotec). Medium was changed every other day. GM-CSF- or M-CSF-differentiated macrophages (GM-Mϕ/M-Mϕ) were stimulated for another 40 h or as indicated with 1 μg/ml LPS (Sigma–Aldrich) and 20 ng/ml human recombinant IFN-γ or 200 ng/ml human recombinant IL-10 (both from Miltenyi Biotec), respectively. All cytokines and growth factors were dissolved in endotoxin-free water (Sigma–Aldrich).

For particle uptake experiments, monocytes were cultured in petri dishes (Ø60 mm) at a density of 6 × 10^6^ cells per dish for 4 days at 37°C and 5% CO_2_ in Macrophage-SFM (Life Technologies) supplemented with GM-CSF or M-CSF as described above. On day 4, cells were detached from plates using PBS supplemented with 5 mM EDTA (Sigma–Aldrich) and seeded into 24-well plates at a density of 1.5 × 10^5^ cells/well. On the next day, cells were stimulated with LPS/IFN-γ or IL-10 as described above. In all experiments comparing GM-MΦ and M-MΦ, cells were generated from monocytes obtained from the same donor.

#### Human Alveolar and Tumor-Associated Macrophages (AM/TAM)

Alveolar macrophages (AM) and tumor-associated macrophages (TAM) were isolated from human non-tumor lung tissue or the respective tumor tissue obtained from patients undergoing lung resection. The use of human material was reviewed and approved by the local ethics committee (State Medical Board of Registration, Saarland, Germany; permission no. 213/06). The informed consent of all participating subjects was obtained.

For TAM isolation, tumor tissue was enzymatically digested using a commercially available enzyme mix optimized for the digestion of human tumors (human tumor dissociation kit, Miltenyi Biotec). Additionally, mechanical dissociation was performed before and during the digestion procedure using the gentleMACS Octo Dissociator according to the manufacturer’s instructions. Cells were washed, resuspended in AM/TAM medium (RPMI 1640, 5% FCS, 100 U/ml penicillin G, 100 μg/ml streptomycin, 2 mM glutamine, Sigma–Aldrich) and incubated at 37°C and 5% CO_2_ for 0.5 h. Adherent cells were thoroughly washed with PBS (137 mM NaCl, 2.7 mM KCl, 10.1 mM Na_2_HPO_4_, 1.8 mM KH_2_PO_4_, pH 7.4), detached with accutase (Sigma–Aldrich), and cultivated at a density of 0.5 × 10^6^ cells per well in a 12-well plate for 2–3 days.

Alveolar macrophages isolation was performed according to a previously described method (Hoppstädter et al., 2010, 2012) with minor modifications. After visible bronchi were removed, the lung tissue was chopped and washed with 100–200 ml PBS. Washing buffer was collected and AM were obtained by centrifugation. Remaining erythrocytes were lysed by incubation with hypotonic buffer (155 mM NH_4_Cl, 10 mM KHCO_3_, 1 mM Na_2_EDTA). After washing and centrifugation, the cell pellet was mock-digested and cells were seeded as described for TAM.

#### THP-1 Cells

THP-1 cells were grown in RPMI 1640 medium supplemented with 10% FCS, penicillin (100 U/ml)/streptomycin (100 μg/ml) and 2 mM glutamine as described previously (Kiemer et al., 2009; Hoppstädter et al., 2012). Cells were differentiated by adding PMA (30 ng/ml, Sigma–Aldrich). After 48 h, cells were stimulated with LPS/IFN-γ or IL-10 as described for MDM.

### Particle Synthesis and Characterization

#### Nanoparticle Synthesis

Fluorescent silica nanoparticles were prepared according to a procedure described previously (Schumann et al., 2012). In brief, 25 nm particles (FD25) were synthesized by l-arginine catalyzed hydrolysis of tetraethoxysilane (TEOS, Sigma–Aldrich) in a biphasic water/cyclohexane system. Addition of Atto647N (Atto-Tec) conjugated with (3-aminopropyl)triethoxysilane (APTES, ABCR) and cysteic acid yielded fluorescent nanoparticles with a mean diameter of ∼25 nm. Further regrowth of these particles yielded fluorescent silica nanoparticles with a mean diameter of ∼41 nm (FD45). All particles were dialyzed against ultrapure water, filtered through 0.2 μm cellulose acetate membranes and stored in sterile containers prior to the biological experiments to ensure both sterility and the absence of pyrogens (Kucki et al., 2014).

#### Transmission Electron Microscopy (TEM)

A CM 200 FEG microscope (Philips) transmission electron microscope (TEM) was used to study particle size and morphology. Samples were prepared by immersion of a 200 mesh carbon-coated copper grid into the undiluted nanoparticle suspension. TEM images were recorded on dried samples (12–24 h) and analyzed using ImageJ software from the National Institutes of Health (http://rsb.info.nih.gov/ij/) to estimate the mean particle size and particle size distribution.

#### Hydrodynamic Diameter in Ultrapure Water

Dynamic light scattering (DLS) measurements were performed at 25°C using a Zetasizer Nano ZS (Malvern Instruments) and a nanoparticle size analyser NPA (Nanotrac). Prior to measurements, all particle suspensions were diluted with ultrapure water (1:10 sample:water). The fitting of correlation data was performed using proprietary Malvern or Nanotrac software. Hydrodynamic diameters represent the mean of three sets of at least 10 sequentially performed measurements. Diameters were derived by Gauss fitting of volume-based particle size distributions using Origin 9.1 (Originlab).

#### Zeta Potential

The zeta potential was measured at 25°C using a Zetasizer Nano ZS (Malvern Instruments). Sample conductivity was adjusted by addition of diluted (0.01 M) potassium chloride solution. Each value represents the mean of three sets of at least 10 sequentially performed measurements.

#### Elemental Analysis (ICP-OES)

The Si content was determined by ICP-OES measurements (Ultima 2, Horiba Jobin Ivon) of the aqueous particle suspensions (wavelength Si: 251.611 nm). Samples were diluted in ultrapure water (1:1,000, v/v) prior to injection *via* a seaspray vaporizer (pressure: 2.64 bar, flow rate: 1.04 l/min).

#### Spectroscopic Characterization and Particle Leaching

UV-Vis spectra were recorded using a Cary300Scan UV (Varian) UV-Vis spectrometer for non-diluted samples in the range from 300 to 800 nm to determine the excitation maximum of the fluorescent particles. A Spex FluoroMax-3 fluorescence spectrometer was used to record fluorescence spectra of diluted samples (1:100, v/v). Leaching of non-covalently bound or loosely adsorbed fluorescent dye at the particle surface and particle matrix was investigated by extensive dialysis of the particles against ultrapure water for at least 3 days. The ratio between initial maximum fluorescence intensity at emission maximum and the particles after the experiment was used to calculate the degree of dye leaching present in the sample.

### Determination of Cell Viability

The MTT [3-(4,5-dimethyl-thiazol-2-)-2.5-diphenyl tetrazolium bromide] colorimetric assay was used to ensure the usage of non-toxic nanoparticle concentrations as described previously (Diesel et al., 2013; Astanina et al., 2014; Ziaei et al., 2015) Briefly, culture medium was replaced by MTT solution (0.5 mg/ml in culture medium) after 24 h of nanoparticle exposure in medium containing 5% FCS. After incubation for 2 h, the MTT solution was removed and cells were solubilized in dimethyl sufoxide (DMSO). Absorbance measurements were performed at 550 nm with 630 nm as the reference wavelength using a microplate reader (Tecan Sunrise). The cell viability index was calculated relative to the untreated control and obtained from at least two independent experiments.

### Flow Cytometry

#### Expression of Intracellular and Surface Markers

Monocyte-derived macrophages were harvested using PBS containing 5 mM EDTA (Sigma–Aldrich). Cells were resuspended in MACS Buffer (PBS pH 7.2 containing 2 mM EDTA, 0.5% (w/v) BSA, and 0.09% (w/v) NaN_3_, Miltenyi Biotec). All antibodies were obtained from Miltenyi Biotec and used at concentrations recommended by the supplier. FcR receptors were blocked using FcR Blocking Reagent (Miltenyi Biotec). Cells were stained with the following antibodies: anti-CD14 (PE, clone TÜK4), anti-CD80 (PE, Clone 2D10), anti-HLA-DR, DP, DQ (FITC, clone REA332), anti-CD163 (FITC, clone GHI/61.1). Cells were analyzed using FACSCalibur (BD Biosciences) and FlowJo software. Results are reported as relative geometric mean of fluorescence intensity (GMFI; GMFI of specifically stained cells related to GMFI of isotype controls).

To detect intracellular CD68 in AM and TAM, the washed cells were fixed for 10 min in 1% (w/v) paraformaldehyde in PBS, pH 7.6, and then stained with anti-CD68 (PE, clone Y1/82A) in saponin buffer (PBS containing 2.5% (v/v) bovine calf serum, 0.05% (w/v) NaN_3_, and 0.2% (w/v) saponin) after permeabilization for 10 min in saponin buffer and blocking for 30 min in 10% (v/v, diluted in saponin buffer) human AB serum (PAA).

#### Particle Uptake

All particle treatments were performed in medium containing 5% FCS, as silica nanoparticles exposed to cells in the absence of serum display a stronger adhesion to the cell membrane, a higher internalization efficiency and increased toxicity (Lesniak et al., 2012). In fact, the absence of proteins does not reflect the *in vivo* situation. After incubation for 1 h with nanoparticles (50 μg/ml) or 1.75 μm microparticles (Fluoresbrite carboxylated YG microspheres, Polysciences, 100 particles/cell), macrophages were washed two times with PBS and detached from plates using PBS containing 5 mM EDTA. Cells were resuspended in MACS buffer and examined on a FACSCalibur (BD Biosciences). Results were analyzed using FlowJo software and are presented as relative GMFI (mean fluorescence intensity of particle-loaded cells related to mean fluorescence intensity of untreated controls).

### RNA Isolation, Reverse transcription, and Real-Time RT-PCR

Total RNA was extracted using the RNeasy plus mini kit (Qiagen). 200 ng of total RNA were reverse transcribed in a total volume of 20 μl using the High-Capacity cDNA Reverse Transcription Kit (Applied Biosystems) according to the manufacturer’s instructions. The CFX96 Touch^TM^ Real-Time PCR Detection System (Bio-Rad) was used for real-time RT-PCR. Primers and dual-labeled probes were obtained from Eurofins MWG Operon. Primer and probe sequences were described previously (Hoppstädter et al., 2010). Standards, from 60 to 0.00006 attomoles of the PCR product cloned into pGEMTeasy (Promega), were run alongside the samples to generate a standard curve. All samples and standards were analyzed in triplicate. The PCR reaction mix consisted of 10x PCR buffer (GenScript), either 2 or 8 mM dNTPs (GenScript), 3–9 mM Mg^2+^, 500 nM sense, and antisense primers, either 2.5 or 1.5 pmol of the respective dual-labeled probe, and 2.5 U of Taq DNA Polymerase (GenScript) in a total volume of 25 μl as described in (Hoppstädter et al., 2010, 2012; Hahn et al., 2014). The reaction conditions were 95°C for 8 min followed by 40 cycles of 15 s at 95°C, 15 s at a reaction dependent temperature varying from 57 to 60°C, and 15 s at 72°C.

### Fluorescence Microscopy

Cells were seeded into a SensoPlate^TM^ 24-well glass-bottom plate (Greiner Bio-One) at a density of 1.5 × 10^5^ cells/well in 1 ml Macrophage-SFM supplemented with the respective cytokines and allowed to adhere overnight. Subsequently, cells were incubated with nanoparticles at a concentration of 50 μg/ml or 1.75 μm microparticles (Fluoresbrite carboxylated YG microspheres, Polysciences) at a ratio of 100 particles per cell in medium containing 5% FCS. After 3 h at 37°C, 5% CO_2_, cells were washed three times with ice cold PBS and fixed with fixing solution (Cell Biolabs) for 15 min at room temperature. Subsequently, cells were washed three times with PBS again and nuclei were counterstained with DAPI (Cell Biolabs) in PBS for 10 min. Cells were kept in PBS for microscopy analysis and stored at 4°C. Particle uptake was analyzed with an Axio Observer Zeiss microscope (Carl Zeiss, Göttingen, Germany) equipped with a MRM Axiocam at a 63× magnification using AxioVision software.

### Statistical Analysis

In general, each experiment was performed at least three times. Data are presented as means + SEM. All data were distributed normally, as determined by the Shapiro–Wilk test. Means of two groups were compared with non-paired two-tailed Student’s *t*-test. Means of more than two groups were compared by one way ANOVA with Bonferroni’s *post hoc* test. Statistical significance was set at a *p*-value of <0.05, <0.01, or <0.001. Data analysis was performed using Origin software (OriginPro 8.6G; OriginLabs).

## Results

### Polarization of Human Monocyte-Derived Macrophages

Macrophages differentiated from monocytes by GM-CSF- or M-CSF-treatment (GM-MΦ/M-MΦ) were stimulated with LPS/IFN-γ or IL-10, respectively, to induce an M1 or M2 phenotype.

**FIGURE 1:**
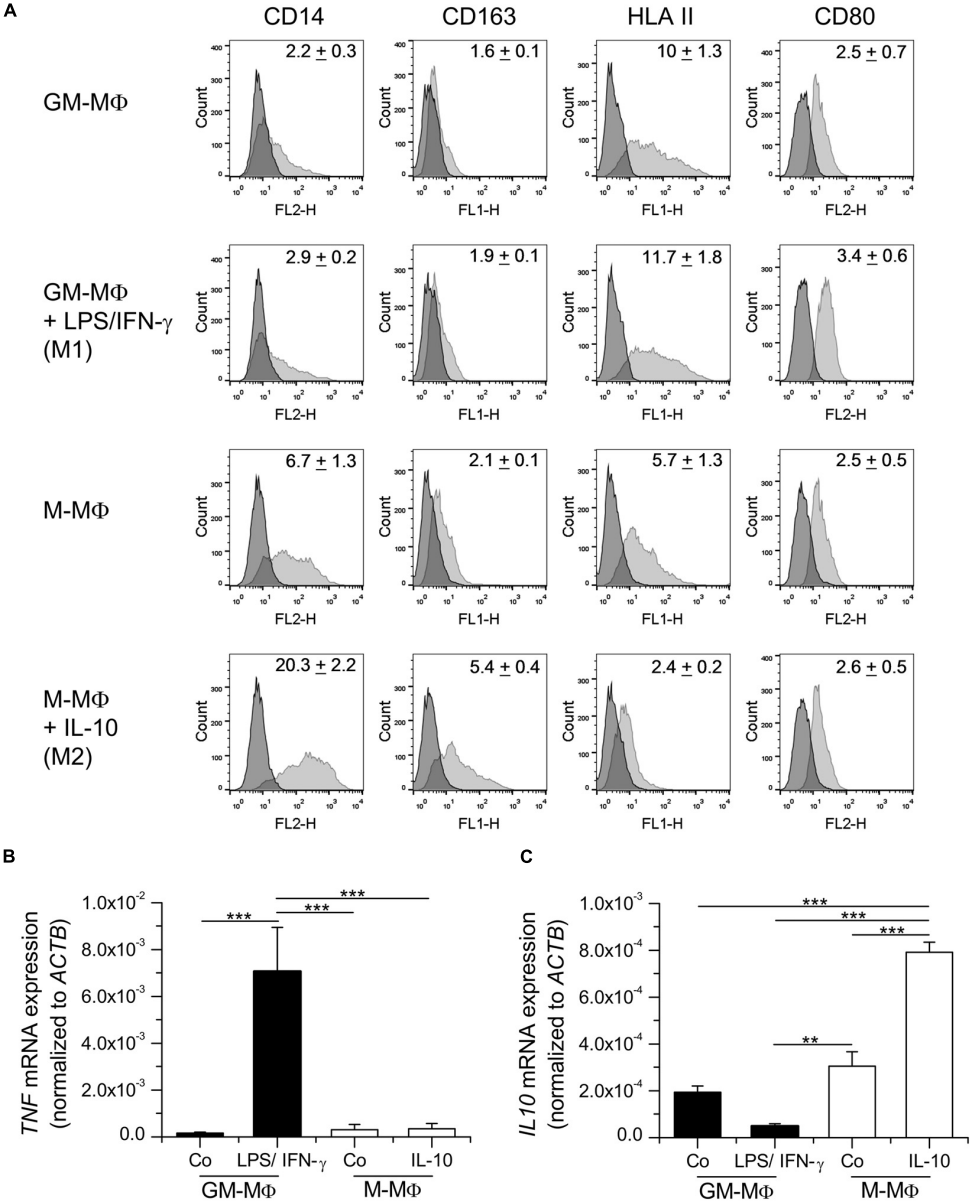
**Characterization of *in vitro* polarized macrophages. (A)** Monocyte-derived macrophages (MDM) were either differentiated with GM-CSF (GM-MΦ) or M-CSF (M-MΦ) and treated with LPS and IFN-γ (M1) or IL-10 (M2), respectively. **(A)** Surface expression of CD14, CD163, HLAII, and CD80 was analyzed by flow cytometry. Dark gray: isotype control; light gray: specific staining. One representative histogram is shown. Geometric mean of fluorescence intensity (GMFI) values are given within graphs and represent the means ± SEM obtained from three independent experiments with cells originating from different donors. **(B,C)** GM-MΦ and M-MΦ were left untreated or treated with LPS/IFN-γ for 4 h or IL-10 for 16 h as indicated. RNA was isolated and the expression of *TNF* **(B)** and *IL10* **(C)** mRNA was quantified by real-time RT-PCR and normalized to *ACTB*. Data show means + SEM of independent experiments performed in duplicate with cells from three different donors. *p*-values were generated by one way ANOVA with Bonferroni’s *post hoc* test. **p < 0.01, ***p < 0.001.

As reported in the literature (Rey-Giraud et al., 2012), GM-MΦ were characterized by high expression of HLAII, low expression of CD14, and the absence of CD163. Further polarization with LPS/IFN-γ resulted in increased CD80 and HLAII surface expression. M-MΦ expressed higher levels of CD14 and CD163 than GM-MΦ or LPS/IFN-γ-treated GM-MΦ. The addition of IL-10 to M-MΦ led to higher expression of CD14 and CD163, whereas HLAII and CD80 were only slightly expressed (**Figure 1A**).

Analysis of cytokine mRNA expression revealed that levels of *TNF* mRNA were elevated in LPS/IFN-γ treated GM-MΦ, correlating with the pro-inflammatory phenotype of these macrophages (**Figure 1B**). On the other hand, *IL10* mRNA was induced by IL-10 treatment in M-MΦ, suggesting a positive feedback loop (**Figure 1C**).

Taken together, both surface marker and cytokine expression were in accordance with reported data on MDM phenotypes induced by LPS/IFN-γ (M1) or IL-10 (M2, also referred to as M2c; Staples et al., 2007; Mosser and Edwards, 2008; Rey-Giraud et al., 2012; Jones et al., 2013; Mills and Ley, 2014).

### Nanoparticle Characterization and Toxicity

**FIGURE 2:**
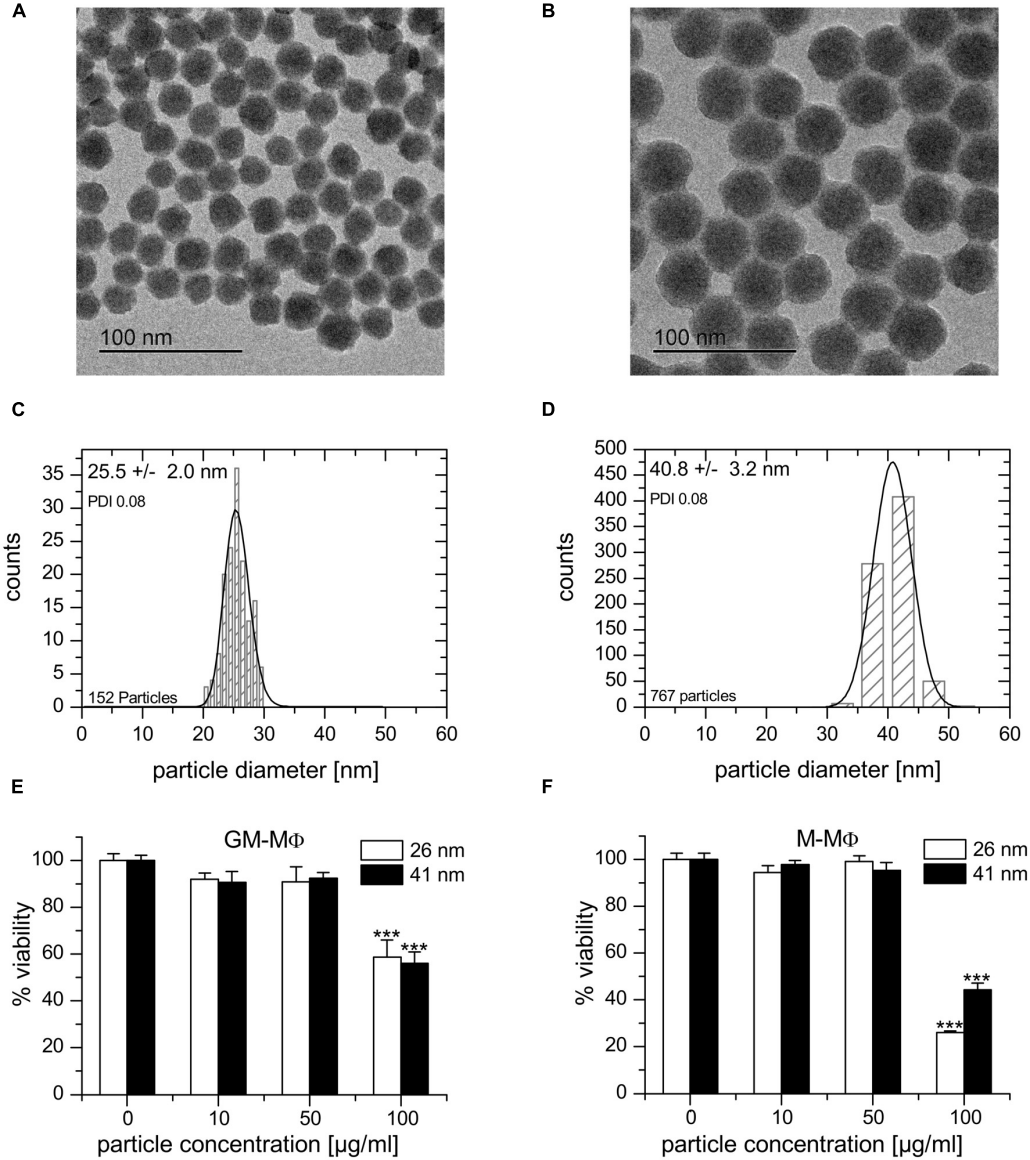
**Nanoparticle characterization and cytotoxicity. (A,B)** TEM image of 25.5 ± 2.0 nm (FD25, A) and 40.8 ± 3.2 nm (FD45, B) fluorescent silica particles. **(C,D)** Size distribution of FD25 **(C)** and FD45 **(D)** nanoparticles. **(E,F)** Cell viability upon nanoparticle exposure as determined by MTT assay. GM-MΦ **(E)** or M-MΦ **(F)** were treated with nanoparticles for 24 h at the indicated concentrations. Data represent means + SEM from two independent experiments performed at least in quadruplicate with cells originating from different donors. Values obtained for untreated cells were set as 100%. *p*-values were calculated by one way ANOVA with Bonferroni’s *post hoc* test. ***p < 0.001, compared with untreated cells.

Particle characterization data are summarized in **Table 1**. Both suspensions exhibited a similar and narrow size distribution (PDI_TEM_ ∼ 0.08) derived from TEM (FD25: 25.5 ± 2 nm, FD45: 40.8 ± 3.2 nm, **Figures 2A–D**) and light scattering experiments (FD25: 24.2 ± 5.5 nm, FD45: 39.1 ± 8.3 nm). Additionally, a similar zeta potential of -30 mV (FD25: -31.2 mV, FD45: -32.3 mV) was observed in aqueous suspension. Based on the ICP-OES derived Si content of suspensions and the TEM derived mean diameter, the particle number concentration of the stock suspensions was calculated to be 833 nM (FD25) and 138 nM (FD45). Both suspensions exhibited identical spectroscopic properties and could efficiently be excited at λ_Ex_ = 647 nm with an emission maximum at λ_Em_ = 660. The particles exhibited only a low degree of dye leaching (<5–10%) and could easily be purified by dialysis against ultrapure water.

**Table 1:** Nanoparticle characterization data.

Silicon oxide NPs	Sample	
	FD 25	FD 45
Particle size (nm)	25.5 ± 2	40.8 ± 3.2
Hydrodynamic diameter (nm)	24.2 ± 5.5	39.1 ± 8.3
Zetapotential (mV)	-31.2	-32.3
Particle concentration (nmol/l)	833	138
λ_Ex_/λ_Em_ (nm)	647/661	647/661
Fluorescence label	Atto647N	Atto647N
Covalently attached	>90%	>95%
Surface	-Si-O-H	-Si-O-H

From measurements using similar nanoparticle preparations, it is known that the zeta potential is reduced in presence of salt ions and even further in presence of serum by formation of a protein corona. In addition, the presence of serum impairs the determination of the hydrodynamic nanoparticle diameter of small nanoparticles (Astanina et al., 2014). According to measurements using larger nanoparticles, the tendency to form large agglomerates increases with increasing particle concentration (unpublished data). At the concentrations applied in this study, particles are not expected to agglomerate extensively.

Viability tests employing the MTT assay showed no significant cytotoxicity of both nanoparticle preparations on GM-MΦ, M-MΦ, THP-1 macrophages, and AM in concentrations up to 50 μg/ml (**Figures 2E,F** and data not shown). Controls for unspecific interactions of nanoparticles with the assay were performed as previously described (Diesel et al., 2013; Astanina et al., 2014).

### Uptake of Nanoparticles and Microparticles in *in vitro* Polarized Macrophages

**FIGURE 3:**
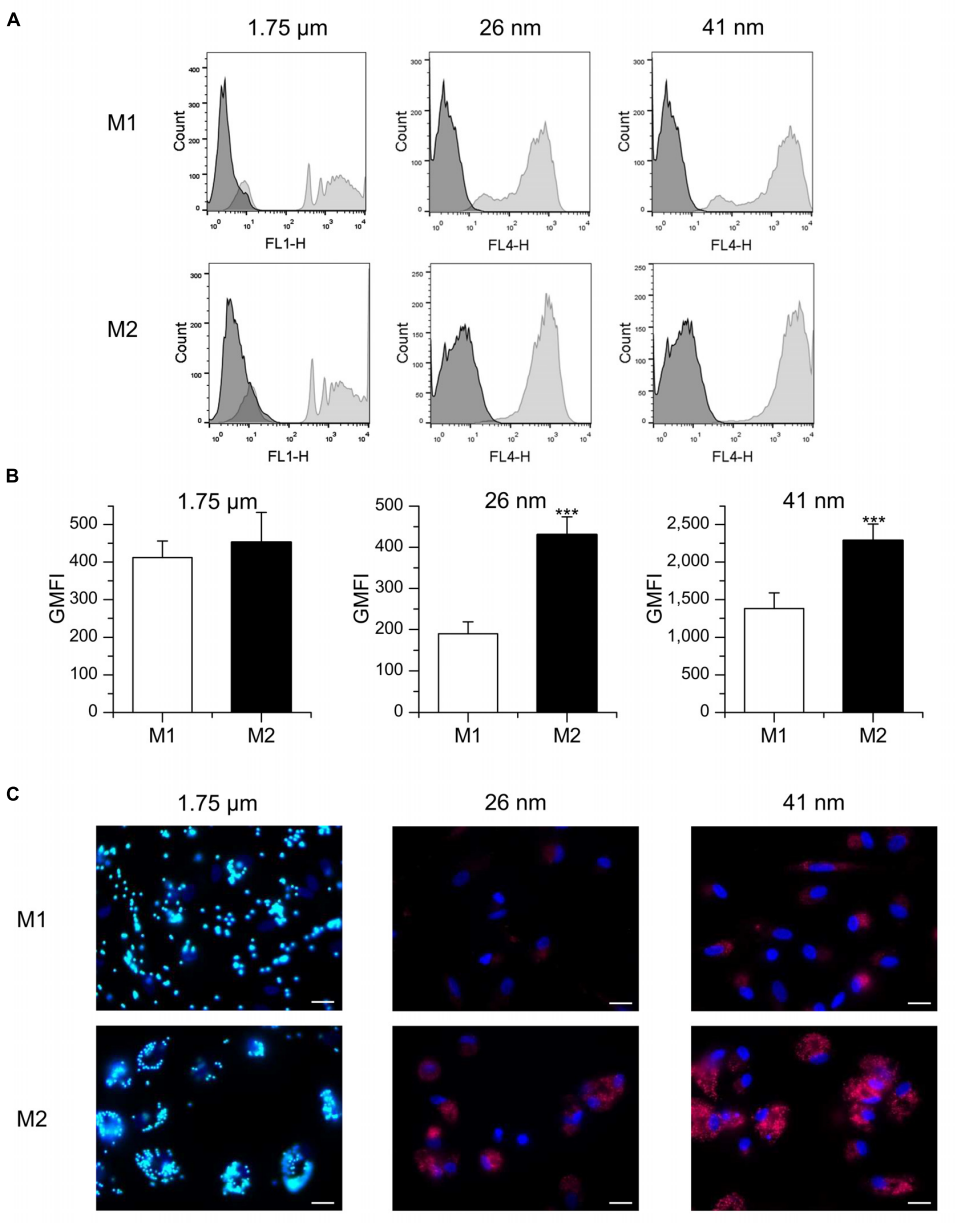
**Particle uptake in M1- and M2-polarized MDM. (A,B)** Macrophages were incubated for 1 h with FITC-labeled 1.75 μm latex beads (100 beads/cell) or fluorescent nanoparticles (26 or 41 nm, 50 μg/ml) and uptake efficiency was assessed by flow cytometry. **(A)** Representative histograms are given. **(B)** GMFI values. Data represent means + SEM of three independent experiments performed in duplicate or triplicate with cells derived from different donors. *p*-values were generated by Student’s *t*-test. ***p < 0.001 compared with M1-polarized cells. **(C)** Representative images of M1 and M2 macrophages 3 h after particle addition. Green: microparticles, red: nanoparticles, blue: nucleus, scale bar: 20 μm.

Particle uptake by M1 and M2 polarized primary human MDM was assessed by flow cytometry (**Figures 3A,B**). M1 and M2 cells internalized 1.75 μm microspheres to a similar extent, as shown by comparable values for relative GMFI. Likewise, no significant difference was observed between M1 and M2 cells regarding the percentage of macrophages positive for particle-associated fluorescence (63.8 ± 5.1% for M1 vs. 69.6 ± 3.3% for M2). In contrast, both M1 and M2 macrophages were >98% positive for particle-associated fluorescence after incubation with nanoparticles. GMFI values were significantly higher in M2 macrophages compared with M1 polarized cells, indicating that both 26 and 41 nm silica particles were taken up more efficiently in M2 cells. Visualization of particle uptake by fluorescence microscopy further confirmed these assumptions and indicated that nanoparticles were in fact localized inside the cells and not merely attached to their surface (**Figure 3C**).

**FIGURE 4:**
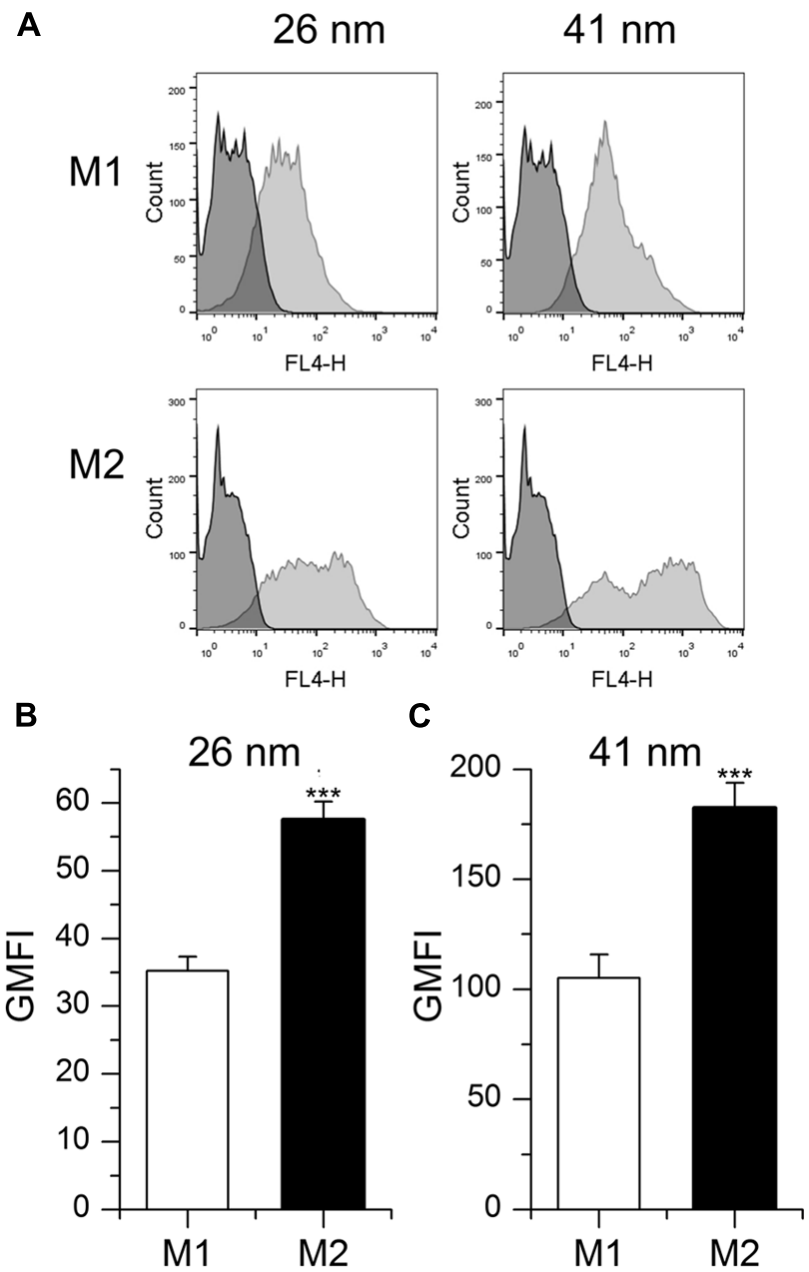
**Particle uptake in M1- and M2-polarized THP-1 macrophages. (A,B,C)** Cells were incubated for 1 h with fluorescent nanoparticles (26 or 41 nm, 50 μg/ml) and uptake efficiency was assessed by flow cytometry. **(A)** Representative histograms are given. **(B,C)** GMFI mean values + SEM of three independent experiments performed in triplicate. *p*-values were generated by Student’s *t*-test. ***p < 0.001 compared with M1-polarized cells.

In addition to MDM, the macrophage-like cell line THP-1 is widely used to investigate the impact of M1 and M2 polarization on distinct cell functions (Tjiu et al., 2009; Chanput et al., 2013). Therefore, we also analyzed nanoparticle uptake in these cells after treatment with LPS/IFN-γ or IL-10 to induce an M1 or M2 phenotype, respectively. As observed in primary MDM, the uptake potential for nanoparticles was increased in M2-polarized THP-1 macrophages when compared with M1 cells, as suggested by significantly increased GMFI values (**Figure 4**).

### Nanoparticle Uptake in Primary Human Alveolar Macrophages and Tumor-Associated Macrophages

**FIGURE 5:**
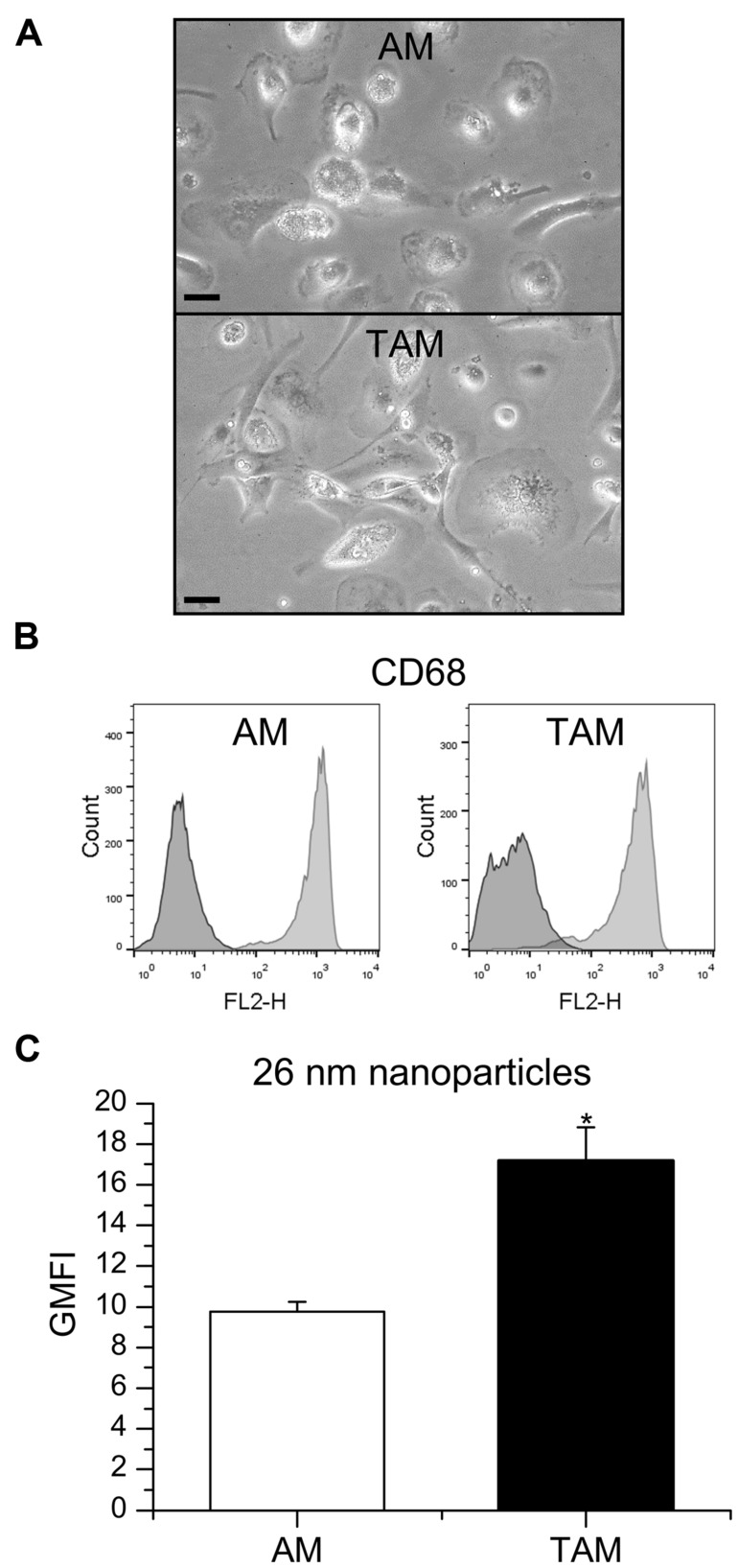
**Nanoparticle uptake in AM and TAM. (A)** AM and TAM morphology were examined by light microscopy. One representative image is given. Scale bar: 20 μm. **(B)** CD68 expression in AM and TAM. Data show one histogram representative for four independent experiments. Dark gray: isotype control; light gray: specific staining. **(C)** GMFI mean values + SEM obtained from independent experiments with AM obtained from two and TAM obtained from four different donors. *P*-values were calculated by Student’s *t*-test. *p < 0.05 compared with AM.

In general, TAM represent M2-like macrophages promoting tumor cell proliferation, angiogenesis, matrix turnover, and repression of adaptive immunity (Solinas et al., 2009). In contrast, AM are considered to exhibit a more pro-inflammatory, M1-like phenotype (Hoppstädter et al., 2010). Therefore, we hypothesized that the capacity to take up nanoparticles might differ between those two cell types. TAM were obtained after digestion of tumor tissue from patients undergoing lung resection, whereas AM were isolated from the surrounding non-tumor lung tissue. AM populations mostly consisted of large, round cells whereas TAM were more heterogenous in size and shape (**Figure 5A**). Intracellular CD68, often used as a marker specific for macrophages (Holness and Simmons, 1993; Hoppstädter et al., 2010), was detected in over 95% of the cells contained in AM and TAM preparations, thereby identifying them as macrophages (**Figure 5B**). The uptake of 26 nm silica particles was indeed enhanced in TAM when compared to AM, as assessed by flow cytometry (**Figure 5C**), suggesting that our findings for *in vitro* polarized macrophages also translate to the *in vivo* situation.

## Discussion

The use of silica-based nanomaterials in commercial products, e.g., as additives to food, cosmetics, varnishes, or printer toners, is rapidly increasing. In addition, silica or silica coated engineered nanoparticles have been suggested as promising candidates for biomedical applications, such as gene transfection, drug delivery, biosensing, and imaging applications (Ravi Kumar et al., 2004; Knopp et al., 2009; Rosenholm et al., 2010; Probst et al., 2012; Korzeniowska et al., 2013; Montalti et al., 2014). The growing commercialization of nanotechnology products has raised concerns about their safety. The physico-chemical properties of silica nanoparticles that make them attractive for industrial use might represent potential hazards to human health, due to an enhanced ability to penetrate tissues or even cells and their interactions with biomolecules. Investigations on their potential to induce cell death or inflammation led to divergent results. Apart from the composition and size of nanomaterials, the target cell type critically affects intracellular responses and the degree of cytotoxicity (Napierska et al., 2010; Sohaebuddin et al., 2010; Izak-Nau et al., 2013).

After entering the body, nanoparticles are rapidly cleared by macrophages and other cells of the mononuclear phagocyte system (MPS; Yoo et al., 2010; Amoozgar and Goldberg, 2014). Besides tissue macrophages present in every organ of the body, the MPS includes committed precursors in the bone marrow and circulating blood monocytes (Jenkins and Hume, 2014). Nanoparticles entering tissues or circulating in the blood make direct contact with various MPS cells. Previous studies have shown that the MPS is responsible for the clearance of most nanoparticles larger than 10 nm, regardless of their shape and surface chemistry (Longmire et al., 2008).

Nanoparticle uptake by MPS cells can occur through various pathways in macrophages, phagocytosis, and macropinocytosis, as well as clathrin-, caveolae-, and scavenger receptor-mediated endocytic pathways have been suggested to be involved in nanoparticle internalization (Diesel et al., 2013; Kuhn et al., 2014; Roy et al., 2014). Nanoparticle exposure can lead to pro-inflammatory responses, most of which are associated with macrophages. The avid uptake of nanoparticles by these cells might make them more susceptible to particle overload and cell death (Napierska et al., 2010; Sohaebuddin et al., 2010). Thus, the characterization of nanoparticle uptake in macrophages is an important step in the assessment of nanoparticle toxicity.

In the present study, we demonstrated that macrophage polarization influences particle uptake in primary human macrophages and human macrophage-like THP-1 cells. M1 macrophages are considered to be more involved in inflammatory and microbicidal processes, and have been shown to be more phagocytic towards bacteria (Varin et al., 2010; Krysko et al., 2011). In contrast, M2 macrophages are generally thought to exert anti-inflammatory functions and to promote wound healing. They might also be more involved in debris clearance, since they exhibit a greater phagocytic activity towards cell debris compared with M1 (Rey-Giraud et al., 2012), indicating that the influence of macrophage polarization on phagocytosis largely depends on the properties of the phagocytosed material. Accordingly, phagocytosis has been suggested as a general property of macrophages, but not a reliable predictor of M1 or M2 responses (Mills and Ley, 2014). In fact, we did not detect any differences between primary M1 and M2 macrophages regarding the phagocytic uptake of latex microparticles. In line with our findings, microparticle clearance has been reported to be similar in Th1- and Th2-prone mouse strains (Jones et al., 2013).

On the other hand, we observed a markedly increased uptake of both 26 and 41 nm silica nanoparticles following M2 polarization compared to M1 cells in primary as well as THP-1 macrophages. M2 macrophages have been shown to internalize FITC-dextran and 300 nm PEG hydrogel nanoparticles more efficiently when compared to M1 polarized cells, indicating that M2 polarization leads to a higher endocytic capacity (Edin et al., 2013). This might be due to increased expression of receptors facilitating endocytosis, i.e., scavenger and lectin receptors, in M2-polarized cells (Martinez et al., 2006; Rey-Giraud et al., 2012; Jones et al., 2013). Furthermore, the Th1-biased mouse strain C57BL/6 has been reported to clear nanoparticles more slowly than the Th2-prone Balb/c strain, which might be mainly due to the prevalence of M2 macrophages in Balb/c mice (Jones et al., 2013).

The unique physical and chemical properties associated with potentially detrimental effects of nanoparticles on cells and tissues might be beneficial in the context of nanomedicine. In fact, nanomaterials offer many advantages, such as improved bioavailability and feasibility of incorporation of both hydrophilic and hydrophobic substances, and may be used in various biomedical applications ranging from diagnostics to therapeutics (Latterini and Amelia, 2009; Zhao et al., 2009; Chang et al., 2014; Vijayanathan et al., 2014). Due to their hydrophilicity, stability in physiological environment, ease of production, and relatively low cost, silica nanoparticles display a great potential for biomedical applications (Bitar et al., 2012).

However, rapid elimination from the systemic circulation by cells from the MPS constitutes a major challenge for the application of nanoparticles as intravenous drug delivery platforms, as it greatly reduces the number of nanoparticles available at the target site, thereby impairing the efficacy of the drug (Yoo et al., 2010; Amoozgar and Goldberg, 2014). At the same time, nanoparticle accumulation in macrophages has been considered to be an advantage for therapeutic strategies based on macrophage reprogramming towards a stimulatory/destructive or a suppressive/protective phenotype (Chellat et al., 2005).

A recently published meta-analysis revealed that the inter-patient pharmacokinetic variability of nanoparticulate formulations is higher compared with small molecule agents (Schell et al., 2014). The patients’ immune status and thereby their prevailing macrophage phenotype can be influenced by various immune-priming events such as allergies or infections (Sica and Mantovani, 2012). Thus, our data suggest that the macrophage phenotype might contribute to the high inter-individual pharmacokinetic variability of nanoparticulate drugs, with analogous implications for the clearance of potentially harmful nanoparticles taken up from the environment.

We previously reported that distinct macrophage populations residing in the human lung exhibit different phenotypic and functional characteristics: AM resembles inflammatory M1 macrophages, whereas lung interstitial macrophages display a more regulatory phenotype (Hoppstädter et al., 2010). Macrophages are also one of the major populations of infiltrating leukocytes in solid lung tumors. These TAM play an important role in tumor initiation, development, and metastasis. TAM are considered to be a polarized M2-like macrophage population with potent immunosuppressive functions. High numbers of TAM are associated with a poor prognosis, accelerated lymphangiogenesis, and lymph node metastasis (Sica et al., 2008; Solinas et al., 2009; Ma et al., 2010). In the present study, we compared the nanoparticle uptake capacity of human primary TAM from non-small cell lung cancer tissue samples with AM from non-tumor tissue. As observed for *in vitro* differentiated M2 macrophages, the internalization of 26 nm silica nanoparticles was clearly enhanced in TAM. Since TAM retain functional plasticity, reprogramming TAM in order to eliminate their support for tumor growth or to induce cytotoxic activity has been considered as a strategy to improve tumor therapy (Sica et al., 2007; Stout et al., 2009; Chakraborty et al., 2012; Amoozgar and Goldberg, 2014). Considering the high potential for nanoparticle uptake observed in TAM, such therapeutic approaches might benefit from the use of nanoparticulate formulations.

In summary, our data suggest that the interaction of nanoparticles with differentially polarized macrophages should be taken into consideration when investigating the potentially toxic health effects of nanomaterials. What is more, the preferential uptake of nanoparticles by M2-like macrophages might offer new therapeutic approaches aimed at targeting M2 macrophages.

## Author Contributions

JH participated in study design and data acquisition and wrote the manuscript. MS and AD performed all macrophage-related experiments. CC and AK provided nanoparticles and nanoparticle characterization data and participated in manuscript preparation. HH provided lung and tumor tissue, participated in data interpretation, and edited the manuscript. AKK initiated the study and participated in data interpretation and manuscript preparation. All authors read and approved the final manuscript.

## Conflict of Interest Statement

The authors declare that the research was conducted in the absence of any commercial or financial relationships that could be construed as a potential conflict of interest.
